# Supplementary material for: Overexpression of Type 1 and 2 Diacylglycerol Acyltransferase Genes (JcDGAT1 and JcDGAT2) Enhances Oil Production in the Woody Perennial Biofuel Plant Jatropha curcas
Source: Plants (Basel). 2021 Apr 5;10(4):699. doi: 10.3390/plants10040699 (PMC8066779; doi:10.3390/plants10040699)
Supplement: Supplementary file 1 [file plants-10-00699-s001.pdf]

plants

MDPI

Supplementary Material

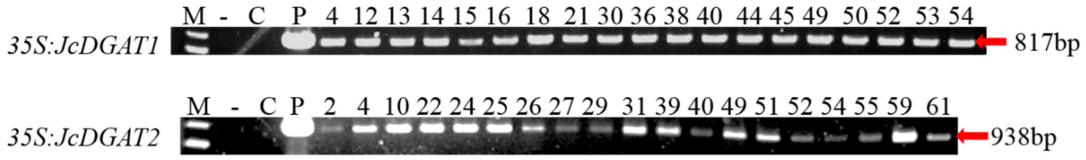

Figure S1. Identification of transgenic *J. curcas* plants by PCR. The amplified fragments contained partial sequences of the *35S* promoter and *JcDGATs* cDNA. Lanes: M, Trans 2 kb DNA ladder; -, blank control without DNA template; C, negative control (control plants); P, positive control (plasmids); numbers, regenerated transgenic lines.

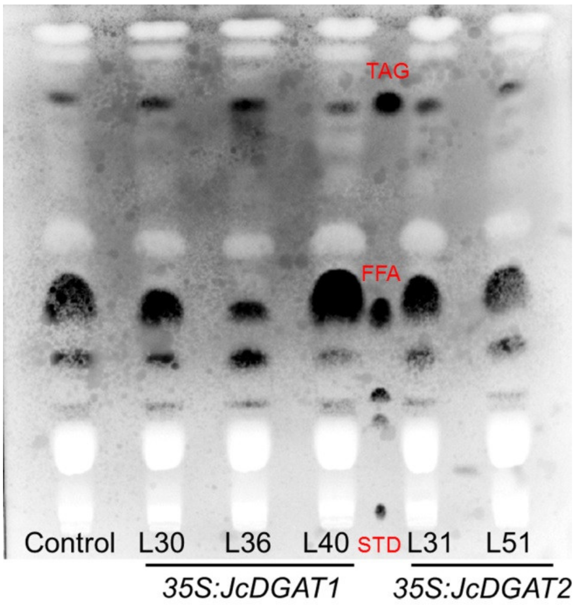

Figure S2. TLC separation of neutral lipids in leaf tissues from control and transgenic *J.curcas* lines. Leaves were sampled during the period of fruiting. STD represents the TLC reference standard. TAG represents triacylglycerol and FFA represents free fatty acid.

Table S1. List of primers used in this study.

Name	Forward	Reverse
JcActin1	5′-CTCCTCTCAACCCCAAAGCCAA-3′	5′-CACCAGAATCCAGCACGATACCA-3′
JcDGAT1	5′-ATTCTCTCTTGCCGCCTATC-3′	5′-CAGAACAGCAGACCCACAAC-3′
JcDGAT2	5′-CTGGTGGAGTTCAAGAGACATT-3′	5′-ACTTGCCATCAGGTTTCCAC-3′
35S:JcDGAT1	5′-TCCACTGACGTAAGGGAT-3′	5′-CAGAACAGCAGACCCACAAC-3′
35S:JcDGAT2	5′-TCCACTGACGTAAGGGAT-3′	5′-ACTTGCCATCAGGTTTCCAC-3′
